# Nutritional Status and Cardiovascular Disease in Older Adults: Clinical Perspectives from Mechanisms to Management

**DOI:** 10.3390/jcm15135064

**Published:** 2026-06-29

**Authors:** Iris Parrini, Carmelo Massimiliano Rao, Fabiana Lucà, Michele Massimo Gulizia, Roberto Ceravolo, Sandro Gelsomino, Nadia Ingianni, Caterina Patrizia Ceruso, Clementina Silvia Iannì, Maria Teresa Ferrò, Claudio Bilato, Giovanna Geraci, Fabrizio Oliva, Federico Nardi, Massimo Grimaldi

**Affiliations:** 1 Cardiology Unit, Koelliker Hospital, 10134 Torino, Italy; 2 Cardiology Unit, Santa Maria Degli Ungheresi Hospital, Polistena, 89024 Reggio Calabria, Italy; 3 Cardiology Department, Grande Ospedale Metropolitano of Reggio Calabria, 89124 Reggio Calabria, Italy; 4 Cardiology Department, Garibaldi Nesima Hospital, 95122 Catania, Italy; 5 Cardiology Unit, Giovanni Paolo II Hospital, 88046 Lamezia Terme, Italy; 6 Cardiothoracic Department, Maastricht University Hospital, 6229 Maastricht, The Netherlands; 7 Cardiologic District, Azienda Sanitaria Provinciale Di Trapani, 91016 Trapani, Italy; 8 Cardiology Unit, N. Giannettasio Hospital, 87064 Rossano, Italy; 9 Cardiology Unit, Cetraro-Paola Hospital, 87027 Paola, Italy; 10 Cardiology Unit, Arzignano Hospital, 36071 Arzignano, Italy; claudio.bilato@aulss8.veneto.it; 11 Cardiology Department, Sant’Antonio Abate Hospital, Erice, 91016 Trapani, Italy; 12 Cardiology Unit, ASST Grande Ospedale Metropolitano Niguarda, 20162 Milano, Italy; fabrizio.oliva@ospedaleniguarda.it; 13 Cardiology Department, Santo Spirito Hospital, Casale Monferrato, 15033 Alessandria, Italy; 14 Cardiology Department, General Regional Hospital “F. Miulli”, Acquaviva Delle Fonti, 70021 Bari, Italy

**Keywords:** nutrition, malnutrition, older adults, CV disease, frailty, Mediterranean diet, sarcopenia

## Abstract

Nutritional status is a pivotal determinant of health and longevity in ageing populations. Malnutrition, overnutrition, and sarcopenic obesity are increasingly recognised as key modifiers of CV risk in older adults. Beyond caloric imbalance, age-related metabolic, hormonal, and inflammatory changes profoundly influence CV structure and function. Both nutrient deficiency and excess contribute to endothelial dysfunction, oxidative stress, and chronic inflammation, core mechanisms underlying atherosclerosis, heart failure, and frailty. This review analyses the complex interplay between nutrition and cardiovascular diseases in older adults, outlining physiological mechanisms, diagnostic tools for nutritional assessment, and evidence-based dietary strategies aimed at prevention and management. Particular attention is given to the challenges of multimorbidity, polypharmacy, and altered body composition that shape nutritional care in geriatric cardiology.

## 1. Introduction

Worldwide demographic ageing has transformed the landscape of cardiovascular disease (CVD). Individuals aged ≥ 65 years now represent more than 20% of the total population in developed countries, and the incidence of CVD, including coronary artery disease (CAD), heart failure (HF), and atrial fibrillation (AF), rises steeply after the seventh decade [1]. Concomitantly, malnutrition, sarcopenia, and obesity coexist in complex forms that aggravate vascular dysfunction and adverse outcomes [2].

Malnutrition in older adults should not be interpreted solely as insufficient caloric intake or low body weight [3,4]. Contemporary cardiovascular (CV) and geriatric research increasingly recognises malnutrition as a multidimensional biological condition that extends beyond simple caloric deficiency. In older adults with CVD, altered body composition, chronic inflammation, anabolic resistance, sarcopenia, and metabolic dysregulation frequently coexist, contributing to reduced functional reserve, frailty progression, and adverse clinical outcomes [4]. This concept encompasses not only undernutrition but also obesity-related nutritional imbalance and sarcopenic phenotypes frequently observed in older patients with CVD.

**Figure 1:**
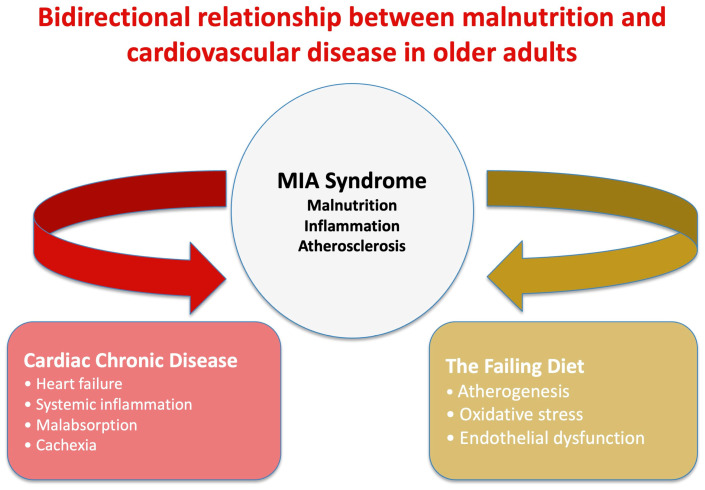
Bidirectional relationship between nutritional status and CVD in older adults. Chronic CVD may promote anorexia, systemic inflammation, intestinal congestion, and malabsorption, contributing to nutritional decline. In parallel, malnutrition and altered nutritional status may accelerate oxidative stress, endothelial dysfunction, inflammation, and progression of atherosclerotic disease, generating a self-reinforcing malnutrition–inflammation–atherosclerosis (MIA) cycle.

The relationship between nutritional status and CVD is bidirectional. On one side, chronic cardiac disease induces anorexia, cachexia, and malabsorption through systemic inflammation and gut oedema [5]; on the other, poor nutritional intake may contribute to atherogenesis, impaired endothelial repair, and reduced immune competence. The so-called “malnutrition–inflammation–atherosclerosis” (MIA) cycle exemplifies this interdependence [6]. This interaction is particularly relevant among older adults, in whom multimorbidity, frailty, and systemic inflammation frequently overlap, complicating both nutritional assessment and CV management (Figure 1).

Given that nutritional impairment in older CV patients is frequently multifactorial and poorly captured by body weight alone, multidimensional assessment tools for nutrition and body composition have emerged as important instruments for risk stratification. Clinical screening scores, including the Mini Nutritional Assessment (MNA/MNA-SF) [7], Geriatric Nutritional Risk Index (GNRI) [8], Controlling Nutritional Status (CONUT) score [9], and Prognostic Nutritional Index (PNI) [10], together with body composition techniques such as bioelectrical impedance analysis (BIA) and dual-energy X-ray absorptiometry (DXA), may improve the identification of sarcopenia, altered adiposity distribution, and “normal-weight malnutrition”, limitations often overlooked by body mass index (BMI) alone [11,12].

Older patients may display “normal-weight malnutrition” with sarcopenia and visceral adiposity despite stable BMI [13,14]. Identifying and managing malnutrition is therefore an essential component of comprehensive CV care.

Recent epidemiological data link low serum albumin, low body weight, and unintentional weight loss to increased mortality after myocardial infarction and in chronic HF [15,16,17]. Conversely, the so-called “obesity paradox,” whereby overweight appears to be associated with lower mortality in selected HF cohorts, remains controversial and may partly reflect BMI’s inability to distinguish among protective lean mass, fluid retention, and dysfunctional visceral adiposity [18]. Accordingly, nutritional phenotyping should be considered an integral component of modern CV care in older adults, moving beyond weight-centred approaches to a more comprehensive evaluation of metabolic health, muscle preservation, frailty, and functional reserve [19,20].

Although malnutrition, cachexia, sarcopenia, frailty, and sarcopenic obesity frequently coexist in older adults with CVD, they represent distinct clinical entities with different pathophysiological mechanisms, assessment strategies, and therapeutic implications. Their main characteristics are summarised in Table 1.

The aim of this review is to critically summarise the current evidence on the complex relationship between nutritional status and CVD in older adults, with a particular focus on malnutrition, sarcopenia, altered body composition, frailty, and contemporary nutritional strategies for CVD prevention and management.

## 2. Methods

### 2.1. Search Strategy and Study Selection

A structured literature review with systematic search methods was conducted to identify and summarise the available evidence regarding nutritional status and CVD in older adults. PRISMA 2020 recommendations were used as a methodological framework to support a transparent and reproducible literature search and study selection process [33].

The search strategy was independently developed by two authors (I.P. and F.L.) and subsequently reviewed and approved by a third investigator (C.M.R.). Additional references identified through the bibliography of retrieved articles were manually screened and cross-checked to identify potentially relevant studies not captured by the initial database search. A systematic literature search was performed in PubMed/MEDLINE and EMBASE from January 2005 to March 2025. Titles and abstracts were screened according to predefined eligibility criteria. Database queries were refined by an investigator experienced in literature search methodology, with particular attention to studies addressing nutritional status, frailty, body composition, multimorbidity, and CVD in older adults. The search strategy was designed to identify studies evaluating the relationship between nutritional status and CVD in older populations, with a specific focus on malnutrition, overnutrition, sarcopenia, sarcopenic obesity, altered body composition, frailty, multimorbidity, and nutritional-care pathways. The following search strategy was used: (“older adults” OR older adults OR geriatric OR aged) AND (“CV disease” OR “heart failure” OR “coronary artery disease” OR “atrial fibrillation” OR “cardiac rehabilitation” OR “cardiac surgery”) AND (malnutrition OR “nutritional risk” OR “nutritional status” OR MNA OR GNRI OR CONUT OR PNI OR MUST OR NRS-2002) AND (obesity OR overnutrition OR “sarcopenic obesity” OR sarcopenia OR “body composition” OR “muscle mass” OR “fat mass” OR BIA OR DXA) AND (multimorbidity OR comorbidity OR frailty OR “nutritional care” OR “nutritional intervention” OR “oral nutritional supplement”). No language restrictions were initially applied during the search process. Observational studies, systematic reviews, and interventional studies were considered eligible for inclusion.

### 2.2. Eligibility Criteria

Studies were considered eligible if they focused on CVD, including HF, CAD, ACS, AF, cardiac rehabilitation, or cardiac surgery, and included older adults, defined as individuals aged ≥65 years. Studies involving mixed-age populations were also considered eligible when the mean or median age of the study population was ≥65 years, when the majority of participants were aged ≥65 years, or when separate data for older adult subgroups were available and could be extracted.

Eligible studies were required to assess nutritional status using validated tools or clinically relevant nutritional parameters, including MNA, MNA-SF, GNRI, CONUT, PNI, MUST, NRS-2002, SGA, BMI, BIA, DXA, or other body composition measures.

Studies were included if they reported at least one clinically relevant outcome, such as mortality, hospitalisation, length of stay, readmission, CV events, frailty, functional decline, quality of life, or implementation of nutritional care interventions.

To improve conceptual integration, extracted data were categorised into six predefined nutritional domains: (1) malnutrition; (2) overnutrition/obesity; (3) sarcopenia or sarcopenic obesity; (4) altered body composition; (5) multimorbidity/frailty; and (6) nutritional care. The main characteristics of the included studies are summarised in Table 2.

### 2.3. Exclusion Criteria

Studies were excluded if they were not focused on older adults, did not include a CV population or CV outcome, lacked nutritional assessment, did not provide extractable clinical data, focused exclusively on non-CV conditions without clear applicability to geriatric cardiology, or consisted of editorials, commentaries, case reports, or purely narrative articles without original or systematically extracted data.

### 2.4. Study Selection Process

A total of 179 records were identified through database searching. After title and abstract screening, 121 records were excluded for failing to meet the predefined inclusion criteria. Thirty articles were retrieved for full-text evaluation. Sixteen studies were subsequently excluded because they included non-older populations, lacked a CV focus, lacked a validated nutritional assessment, lacked clinically relevant outcomes, were narrative designs without extractable data, were case-report designs, or contained overlapping information. Ultimately, 14 studies were included in the qualitative synthesis.

**Figure 2:**
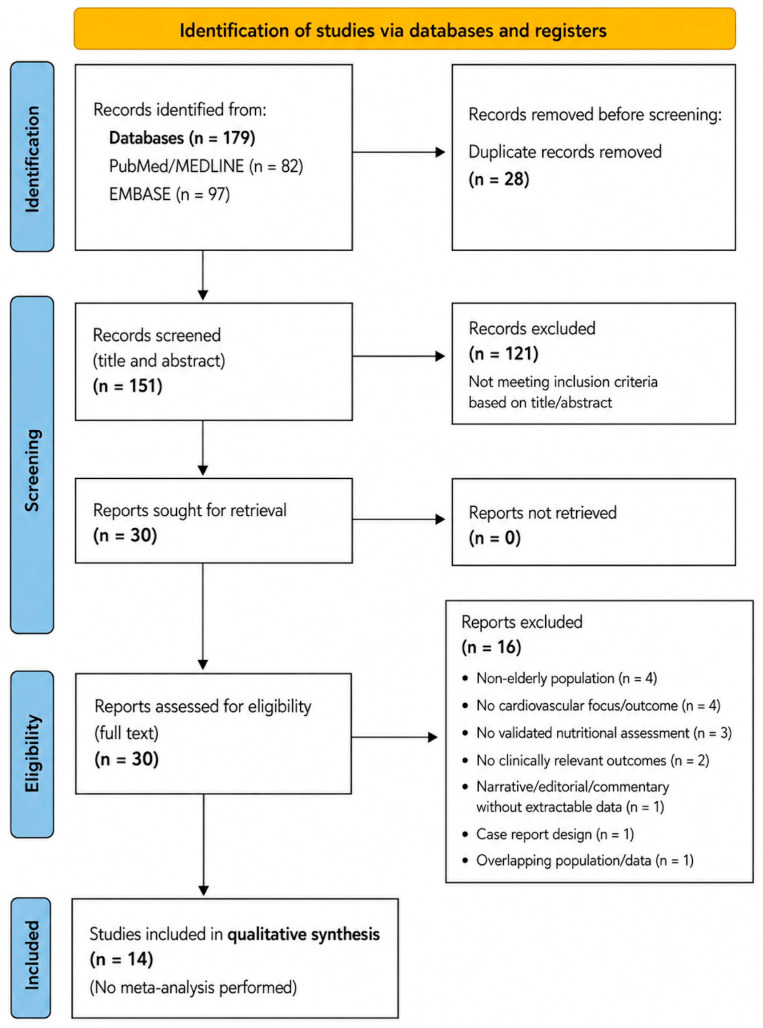
Literature selection process. Flow diagram illustrating the identification, screening, eligibility assessment, and inclusion of studies for the qualitative synthesis.

The study selection process is summarised in Figure 2.

### 2.5. Data Extraction

For each included study, the following variables were extracted: first author, year of publication, study design, population characteristics, sample size, age, CV condition, nutritional assessment tool, body composition measure, definition of malnutrition, obesity, sarcopenia or sarcopenic obesity, frailty or multimorbidity variables, nutritional-care intervention when available, clinical outcomes, and principal findings.

### 2.6. Data Synthesis

Because of substantial heterogeneity in study designs, CV populations, nutritional definitions, body composition assessment methods, and reported outcomes, quantitative pooling and meta-analysis were deemed methodologically inappropriate. Therefore, the findings were synthesised qualitatively using a narrative approach and organised according to both the CV setting and the nutritional domain. The synthesis specifically focused on (1) prevalence and prognostic implications of malnutrition in HF, ACS, CAD, and AF; (2) interactions between malnutrition, frailty, sarcopenia, sarcopenic obesity, and multimorbidity; (3) limitations of body mass index and the emerging role of body composition assessment techniques; and (4) nutritional-care pathways and dietary interventions in geriatric cardiology and cardiac rehabilitation.

## 3. Age-Related Changes and Nutritional Physiology

### 3.1. Body Composition Dynamics

Ageing induces profound alterations in body composition: progressive loss of lean mass, redistribution of fat toward visceral depots, and decline in total body water. Between ages 30 and 80, muscle mass decreases by up to 40%, whereas fat mass may increase by 20–30% [48]. Sarcopenia, defined by reduced muscle mass and strength, is a key driver of frailty and CV vulnerability. Loss of skeletal muscle mitochondrial density and capillary rarefaction may reduce oxidative capacity, thereby promoting insulin resistance and dyslipidaemia [49]. Simultaneously, adipose tissue becomes inflamed and dysfunctional, secreting excess interleukin-6 and tumour necrosis factor-α, which have been associated with endothelial function [50].

These compositional changes profoundly affect pharmacokinetics and haemodynamics [30,45]. Lower plasma volume and albumin levels modify drug distribution and increase exposure to lipophilic CV agents [45,51,52]. Thus, the nutritional phenotype of the older adult heart patient must be interpreted in light of altered physiology, not chronological age alone [30,53].

### 3.2. Metabolic and Endocrine Adaptations

Basal metabolic rate declines by 1–2% per decade after midlife due to loss of lean tissue and decreased mitochondrial activity [54]. Appetite-regulating hormones such as ghrelin, leptin, and cholecystokinin become dysregulated, leading to the so-called “anorexia of ageing” [55]. Concurrently, insulin resistance, declining growth hormone secretion, and reduced testosterone or oestrogen levels contribute to fat accumulation and muscle catabolism [56]. These endocrine shifts not only alter energy balance but also interact with CV homeostasis: leptin resistance promotes hypertension via sympathetic activation, while hyperinsulinaemia enhances sodium retention and vascular stiffness [57].

Inadequate protein intake, common among older adults due to chewing difficulties or cost, exacerbates sarcopenia and worsens cardiac outcomes. Observational studies have associated protein consumption < 1.0 g/kg/day with increased mortality after cardiac surgery and in chronic HF [58]. Conversely, balanced protein distribution (20–25 g per meal) stimulates muscle protein synthesis and preserves function.

### 3.3. Micronutrient Deficiencies and Oxidative Stress

Ageing is accompanied by a decline in the intake and absorption of essential micronutrients, vitamins D, B12, C, and E, folate, selenium, and zinc, which modulate oxidative stress and inflammation [59]. However, current evidence does not support routine supplementation in the absence of documented deficiency or a specific clinical indication [60]. Vitamin D deficiency (<20 ng/mL) is associated with increased arterial stiffness, left ventricular hypertrophy, and HF hospitalisation (HHF) [61]. Low selenium and coenzyme Q10 levels impair antioxidant enzyme activity, contributing to endothelial dysfunction and mitochondrial fatigue [62]. Folate and vitamin B12 deficiencies elevate homocysteine, a recognised risk factor for atherosclerosis and thrombosis [63].

Oxidative stress arises from both nutrient scarcity and excess caloric intake. In older adults, overnutrition activates NADPH oxidase and uncouples endothelial nitric oxide synthase, thereby generating reactive oxygen species that accelerate vascular ageing [64]. Diets rich in fruits, vegetables, and omega-3 fatty acids counteract these mechanisms by providing antioxidants and anti-inflammatory compounds. However, older adults often consume <50% of the recommended daily fibre and antioxidant targets [65].

### 3.4. Gastrointestinal and Renal Changes

Age-related alterations in the gastrointestinal tract and microbiota may impair nutrient absorption and contribute to systemic inflammation, metabolic dysregulation, and CV vulnerability in older adults [66]. Furthermore, reduced renal concentrating ability and thirst perception predispose to dehydration and electrolyte imbalance, aggravating haemodynamic instability in CVD [67].

These gastrointestinal and renal alterations underscore the need for tailored nutrition counselling, including adequate fluid intake and, when appropriate, supplementation. The main age-related physiological and nutritional changes relevant to CV health are summarised in Table 3. Simple screening tools, such as the Mini Nutritional Assessment (MNA) or Subjective Global Assessment (SGA), enable early recognition of nutritional risk in CV outpatients [68].

### 3.5. The Inflammatory–Nutritional Interface

Chronic low-grade inflammation (“inflammageing”) represents a key biological link between poor nutrition and CV pathology [69]. Calorie excess and micronutrient deficiency both activate the nuclear factor-κB pathway, thereby stimulating cytokine release and endothelial dysfunction. Malnutrition, in turn, amplifies inflammation through impaired gut barrier integrity and translocation of bacterial endotoxins [70]. Elevated C-reactive protein and interleukin-6 levels correlate inversely with serum albumin and hand-grip strength, reflecting the bidirectional “nutrition–inflammation” continuum [71]. Addressing nutritional deficits may help attenuate systemic inflammation and may contribute to improved cardiovascular outcomes [72].

### 3.6. Frailty, Malnutrition, and CV Vulnerability

Frailty is a multidimensional clinical syndrome characterised by reduced physiological reserve and increased vulnerability to stressors, resulting in a higher risk of disability, HHF, and mortality in older adults [73]. In CV patients, frailty frequently overlaps with malnutrition, sarcopenia, chronic inflammation, and multimorbidity, creating a complex biological and functional continuum [74]. Malnutrition may accelerate the progression of frailty through muscle wasting, impaired immune function, reduced physical performance, and anabolic resistance, whereas frailty itself may worsen nutritional status due to reduced mobility, fatigue, cognitive impairment, depression, and decreased food intake [75]. Nutritional interventions, combined with physical activity and multidimensional geriatric management, have been associated with the attenuation of functional decline and may improve clinical outcomes in selected populations [76]. However, optimal therapeutic strategies and standardised frailty-oriented nutritional pathways remain insufficiently defined [73].

## 4. Malnutrition, Overnutrition, and CV Risk

### 4.1. Protein–Energy Malnutrition and Cardiac Consequences

Malnutrition remains underdiagnosed in hospitalised and ambulatory older patients. Prevalence ranges from 30 to 50% among those with HF or recent myocardial infarction [77]. Protein–energy deficiency results from decreased intake, absorption, or utilisation of nutrients. In chronic CVD, anorexia is mediated by systemic inflammation, elevated cytokines, and hepatic congestion, which impair appetite and digestion [78].

Cardiac cachexia, defined as ≥5% unintentional weight loss over 6–12 months in the absence of other causes, is a strong predictor of mortality in advanced HF [79]. It reflects a catabolic state driven by increased resting energy expenditure, insulin resistance, and impaired anabolic signalling. Muscle wasting extends beyond the periphery, involving respiratory and myocardial tissues, thereby reducing exercise capacity and left ventricular contractility. Elevated tumour necrosis factor-α and myostatin levels have been associated with lower peak VO_2_ values and poorer survival in observational studies, although the causal contribution of these pathways to adverse outcomes remains incompletely understood [80].

Oral nutritional supplementation with high-protein formulas may improve nutritional status, lean body mass, and functional capacity in selected older patients with HF, frailty, or cardiac cachexia [81]. However, the available evidence remains heterogeneous, and most studies are limited by relatively small sample sizes, short follow-up durations, and variability in nutritional protocols and patient phenotypes [82]. Randomised and rehabilitation-based studies suggest that supplementation providing approximately 400–600 kcal/day and 20–30 g of protein may improve lean mass, exercise tolerance, and 6 min walk distance, particularly when combined with physical rehabilitation or resistance exercise. Nevertheless, robust evidence demonstrating consistent reductions in mortality or HHF remains limited, especially in advanced HF populations with severe multimorbidity or frailty [83]. Oral nutritional supplementation should not be considered a universal intervention for all older adults with CVD. Current evidence suggests that the greatest benefit is observed in selected patients with malnutrition, nutritional risk, frailty, sarcopenia, or cardiac cachexia, particularly when supplementation is integrated into a comprehensive rehabilitation programme [84,85]. Although improvements in nutritional status, body composition, and functional capacity have been reported, evidence supporting consistent reductions in mortality or HHF remains limited [86]. The effectiveness of nutritional supplementation may also vary according to HF phenotype, inflammatory burden, baseline nutritional status, and coexistence of sarcopenia or renal dysfunction, highlighting the need for more individualised and phenotype-oriented nutritional strategies. Small, randomised studies show that supplementation of 400–600 kcal/day with 20–30 g of protein increases lean mass and 6 min walk distance in frail HF patients [87,88].

### 4.2. Obesity, Sarcopenic Obesity, and the “Obesity Paradox”

While undernutrition is detrimental, excessive adiposity also contributes to CVD through insulin resistance, dyslipidaemia, and systemic inflammation. However, in older adults, the relationship between BMI and mortality is U-shaped rather than linear [66]. Observational cohorts reveal that older adults with overweight or mild obesity with HF sometimes exhibit lower mortality than lean counterparts, a phenomenon labelled the “obesity paradox” [89].

**Figure 3:**
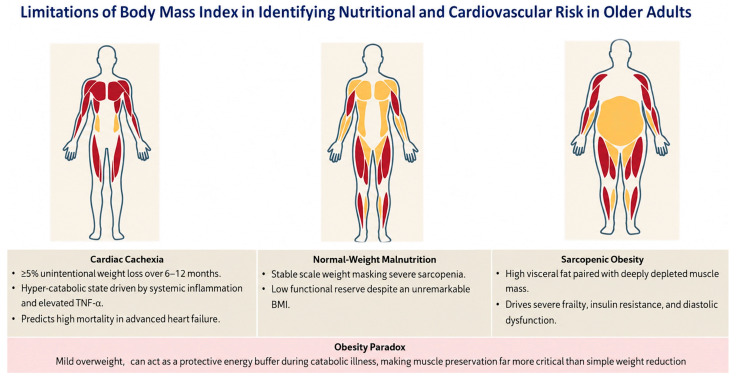
Limitations of body mass index in identifying nutritional and CV risk in older adults.

Several mechanisms may explain this paradox. Fat mass can serve as an energy buffer during catabolic illness, while higher leptin and adiponectin levels may modulate inflammatory pathways (Figure 3). Moreover, BMI poorly differentiates fat from muscle; many “overweight” elders retain greater lean mass and functional reserve [90]. Conversely, “sarcopenic obesity”, high fat but low muscle, has consistently been associated with a less favourable prognosis, including frailty, insulin resistance, and diastolic dysfunction [45,91]. Most evidence regarding the obesity paradox and the prognostic implications of different body composition phenotypes derives from observational studies. Consequently, residual confounding, reverse causality, and limitations of conventional anthropometric measures may partly influence the observed associations [92,93].

In clinical practice, the emphasis should shift from weight loss to optimisation of body composition. Resistance exercise, adequate protein intake (≥1.0–1.2 g/kg/day), and anti-inflammatory diets rich in omega-3 fatty acids may contribute to improvements in body composition and CV risk markers [40].

Body mass index (BMI) alone may fail to accurately reflect the nutritional and metabolic profile of older individuals. Different clinical conditions can present with similar BMI values while concealing markedly different body composition patterns. In cardiac cachexia, progressive unintentional weight loss reflects a hypercatabolic state associated with systemic inflammation and advanced HF and is linked to poor prognosis. Conversely, some older patients may maintain a seemingly normal body weight despite significant loss of skeletal muscle mass, a condition often described as normal-weight malnutrition or hidden sarcopenia, which is associated with reduced functional reserve. At the other extreme, sarcopenic obesity combines excess visceral adiposity with depleted muscle mass, contributing to frailty, metabolic disturbances, and CV dysfunction. These patterns highlight the limited ability of BMI alone to capture clinically relevant alterations in body composition and support the need for a more comprehensive evaluation of nutritional and muscular status in geriatric CV patients [94].

### 4.3. Micronutrient Imbalance and CV Dysfunction

Deficiencies in specific micronutrients have been associated with CV alterations and worse clinical status [95]. Low thiamine levels (<70 nmol/L) impair pyruvate dehydrogenase activity, reducing ATP production and precipitating HF, particularly in patients on chronic diuretics [96]. Iron deficiency, present in 30–50% of HF patients, has been associated with impaired mitochondrial oxidative capacity, anaemia, and fatigue [97]; intravenous iron therapy has been associated with improvements in symptoms and quality of life in selected HF populations [96]. Hypomagnesaemia increases the risk of arrhythmias and sudden death [98], whereas excessive sodium intake has been associated with a higher risk of hypertension and adverse cardiac remodelling, including left ventricular hypertrophy. Balanced micronutrient intake, especially of potassium, magnesium, and calcium, supports vascular tone and electrophysiological stability. Nevertheless, supplementation should be individualised, as indiscriminate multivitamin use may be ineffective or harmful in certain populations [15].

Hypertension and hypertensive heart disease are among the most prevalent CV conditions in older adults and are profoundly influenced by nutritional and metabolic factors. In ageing individuals, arterial stiffness, endothelial dysfunction, visceral adiposity, chronic low-grade inflammation, and altered sodium handling contribute to the development and progression of hypertensive CV remodelling. Nutritional strategies, therefore, represent an important component of CV risk reduction in older hypertensive patients. Current evidence supports Mediterranean-style and DASH-based dietary patterns as the most effective nutritional approaches for blood pressure control and vascular protection in older adults. Adequate intake of potassium, magnesium, calcium, fibre, and antioxidant-rich foods may improve endothelial function and arterial compliance, whereas excessive sodium intake and ultra-processed foods may promote vascular stiffness and left ventricular hypertrophy [99]. However, dietary recommendations in older hypertensive patients should remain individualised according to frailty status, renal function, risk of dehydration or orthostatic hypotension, HF phenotype, and polypharmacy burden, since overly restrictive dietary regimens may worsen sarcopenia, electrolyte imbalance, and functional decline in frail older individuals receiving antihypertensive or diuretic therapies [99].

Routine broad micronutrient supplementation is not supported by current evidence. Instead, replacement strategies should be individualised and primarily considered in the presence of documented deficiency, malnutrition, frailty, impaired absorption, medication-related risk factors, or specific clinical indications [100].

## 5. Assessment of Nutritional Status in the Older Adults

### 5.1. Screening and Diagnostic Tools

**Figure 4:**
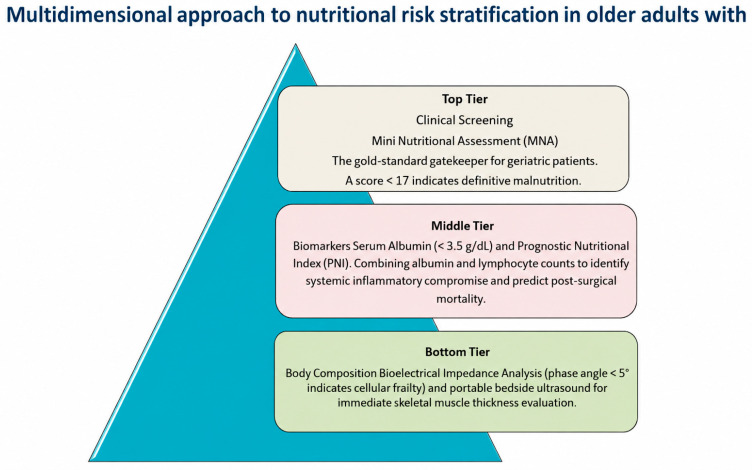
Multidimensional nutritional risk assessment in older CV patients.

Comprehensive nutritional assessment is a cornerstone of CV risk stratification in older adults. The Mini Nutritional Assessment (MNA) remains the most validated screening instrument, combining anthropometric, dietary, and functional indicators. An MNA score < 17 indicates malnutrition, while 17–23.5 denotes risk [67,101]. Other validated tools include the Subjective Global Assessment (SGA), Nutritional Risk Screening (NRS-2002), and Geriatric Nutritional Risk Index (GNRI), which incorporates albumin and body weight [102]. The main nutritional screening and assessment tools are summarised in Table 4 and Figure 4. Biochemical markers may complement clinical nutritional assessment, as serum albumin levels below 3.5 g/dL have been associated with worse outcomes after myocardial infarction and in patients with HF [78,103]. Prealbumin can reflect short-term changes in nutritional status, although it may decrease during inflammatory states. Total lymphocyte counts below 1500/μL may indicate impaired immune function [104] and are measured using the Prognostic Nutritional Index (PNI).

### 5.2. Body Composition Techniques

Anthropometric measures (BMI, waist-to-hip ratio, and mid-arm circumference) remain useful but limited. Bioelectrical impedance analysis (BIA) and dual-energy X-ray absorptiometry (DXA) more accurately quantify fat and lean mass distribution. The phase angle from BIA correlates with cellular integrity and has prognostic value in patients with HF and dialysis [105]. Recent portable ultrasound devices enable bedside measurement of muscle thickness, providing a practical surrogate for assessing sarcopenia [106].

### 5.3. Integration into CV Care

Routine nutritional screening should be incorporated into the initial evaluation of older cardiac patients, alongside blood pressure and lipid profile. In acute settings, such as HHF or ACS, nutritional risk predicts length of stay, infection, and rehospitalisation. Simple interventions, including early referral to a dietitian and tailored meal plans, reduce complications and improve discharge outcomes [15].

Assessment of nutritional status in older individuals with CVD should not rely solely on body weight or body mass index. A comprehensive evaluation requires the integration of clinical screening tools, laboratory biomarkers, and direct measures of body composition. Clinical instruments such as the MNA allow for early identification of patients at risk of malnutrition. Biochemical parameters, including serum albumin and composite indicators such as the Prognostic Nutritional Index (PNI), provide additional information on inflammatory status, immune competence, and overall nutritional reserve. Finally, techniques aimed at evaluating body composition, such as bioelectrical impedance analysis or bedside ultrasound assessment of skeletal muscle thickness, help detect reductions in muscle mass and early manifestations of frailty. The integration of these complementary domains allows for a more accurate characterisation of nutritional vulnerability and supports a more refined risk stratification in older patients with CVD.

## 6. Dietary Patterns and CV Protection

### 6.1. The Mediterranean Model

Among dietary models, the Mediterranean diet is supported by some strong evidence for CV protection [107]. Characterised by high intake of fruits, vegetables, whole grains, legumes, olive oil, and moderate fish consumption, it provides abundant monounsaturated fats, antioxidants, and polyphenols. The PREDIMED trial was associated with a 30% lower incidence of major CV events with the Mediterranean diet supplemented by olive oil or nuts versus a low-fat control [54]. Subgroup analyses reported similar findings and suggested favourable effects among participants aged ≥70 years, with improved endothelial function and lower inflammatory biomarker levels [108].

Adherence to the Mediterranean diet is also associated with slower cognitive decline and lower frailty incidence [109,110]. These associations may be partly explained by mechanisms including the diet’s ability to modulate gut microbiota, reduce oxidative stress, and maintain vascular elasticity. For older patients, practical adaptation includes using extra-virgin olive oil as the primary fat, consuming two servings of vegetables and one of fruit per meal, and ensuring adequate hydration.

### 6.2. DASH and Plant-Based Diets

The Dietary Approaches to Stop Hypertension (DASH) diet, rich in potassium, calcium, and magnesium, effectively lowers systolic BP by 8–14 mm Hg [111]. In older hypertensive individuals, it improves arterial compliance and left ventricular mass index within 3 months. Combination with reduced sodium (<2 g/day) further enhances the benefit [112].

Plant-based and vegetarian patterns, when balanced, lower LDL cholesterol and inflammatory markers. However, strict vegan diets may predispose to B12 and iron deficiency in frail elders unless supplemented [113]. Therefore, partial plant-based regimens such as the “flexitarian” approach may offer the optimal compromise between metabolic health and feasibility.

### 6.3. Protein and Micronutrient Considerations

Adequate protein intake is critical for counteracting sarcopenia and maintaining cardiac function. Current geriatric guidelines recommend 1.0–1.2 g/kg/day, preferably from high-quality sources such as fish, poultry, legumes, and dairy [15]. Distribution throughout the day is essential to maximising muscle protein synthesis. Vitamin D supplementation may be considered in older adults with documented deficiency or an increased risk of deficiency. In these settings, it has been associated with improvements in musculoskeletal health and a lower risk of falls [114]. Omega-3 fatty acids (EPA/DHA 1–2 g/day) may reduce triglyceride concentrations and may provide additional cardiovascular benefits in selected populations, although available evidence remains heterogeneous [115]. Although micronutrient deficiencies are common among older CV patients, evidence supporting routine supplementation to improve CV outcomes remains heterogeneous. Key evidence-based dietary strategies for CV prevention in older adults are summarised in Table 5.

In older adults with CVD, sodium restriction and fluid intake recommendations should be individualised according to frailty status, heart failure phenotype, renal function, risk of dehydration or orthostatic hypotension, and concomitant diuretic therapy. Universal restrictions may not be appropriate for all patients.

### 6.4. Energy Balance and Fluid Intake

Caloric requirements decline with age, but underestimation may worsen frailty. A target of 25–30 kcal/kg/day maintains energy balance in stable elders, with adjustments for disease activity [15]. Fluid intake of 1.5–2 L/day is recommended unless contraindicated; even mild dehydration increases thrombotic risk and orthostatic hypotension [15]. Alcohol should be limited to ≤1 unit/day for women and ≤2 for men, as higher intake increases [116] AF risk [45,66,117,118,119].

### 6.5. Personalisation of Dietary Strategies in Older CV Patients

Dietary interventions in older adults with CVD should be individualised according to comorbidities, functional status, frailty burden, renal function, and risk of malnutrition. In patients with HF and sarcopenia, preservation of skeletal muscle mass and adequate protein intake may represent a priority over aggressive caloric restriction. Conversely, in individuals with obesity with metabolic syndrome or diabetes mellitus, Mediterranean-style dietary patterns combined with moderate caloric control may improve cardiometabolic risk while preserving functional reserve.

Similarly, sodium and fluid recommendations should be adapted according to HF severity, renal function, and risk of dehydration, particularly in frail older adults receiving diuretic therapy. In patients with chronic kidney disease (CKD), nutritional strategies must balance CV protection with the need to avoid excessive intake of protein, potassium, or phosphorus. Cognitive impairment, multimorbidity, polypharmacy, socioeconomic limitations, and reduced appetite should also be considered when planning nutritional interventions in older CV patients.

Therefore, dietary counselling in geriatric cardiology should move beyond standardised recommendations to a multidimensional, patient-centred nutritional approach.

The implementation of nutritional recommendations in older adults with CVD is frequently challenged by factors that extend beyond medical considerations [120]. Polypharmacy may influence appetite, gastrointestinal tolerance, taste perception, and adherence to dietary prescriptions [121]. Cognitive impairment, depression, physical disability, and reduced manual dexterity may further limit the ability to purchase, prepare, or consume nutritionally adequate meals [15]. In addition, social isolation, limited caregiver support, and food insecurity may significantly affect dietary quality, particularly among frail older individuals living alone [15]. Practical solutions should be individualised and integrated into routine CV care [122]. Multidisciplinary collaboration among cardiologists, geriatricians, dietitians, nurses, caregivers, and social support services may facilitate nutritional assessment and long-term adherence [119,122]. Simplified meal plans, adaptation to cultural preferences, and periodic reassessment of nutritional goals may improve feasibility and patient acceptance [66]. Economic aspects should also be considered when implementing nutritional interventions [66]. Although nutritional screening and counselling require dedicated resources, early identification of nutritional vulnerability may help prevent HHF admissions, functional decline, and complications associated with frailty [123]. From a healthcare system perspective, timely nutritional management may therefore represent a cost-conscious strategy within comprehensive CV care for older adults [67].

### 6.6. Dietary Counselling and Adherence

Nutritional intervention succeeds only when adapted to cognitive and functional status. Simplified meal plans, caregiver involvement, and community meal programs improve adherence. Periodic reinforcement by dietitians sustains behavioural change [124,125]. Technology-based tools, apps, and telemonitoring can aid in tracking food intake and weight, supporting long-term compliance even in older populations [126].

## 7. Nutritional Interventions and Clinical Implementation

### 7.1. Individualised Nutritional Therapy

Nutritional intervention in older cardiac patients must begin with a comprehensive assessment that integrates anthropometric measures, comorbidities, and functional capacity. Individualised therapy aims to correct specific deficiencies while maintaining overall energy balance. Calorie-dense but nutrient-rich foods (e.g., fortified dairy, legumes, and whole grains) are preferable to refined carbohydrates. Oral nutritional supplements providing 400–600 kcal/day with at least 20 g of protein improve nitrogen balance and functional recovery in malnourished older adults with HF or post-ACS [15].

Protein and energy supplementation should be combined with resistance or chair-based exercise to optimise anabolic response. In hospitalised elders, early nutritional support within 48 h reduces infection, pressure ulcers, and length of stay [126]. In the outpatient setting, structured dietitian-led programmes reinforce adherence and enable dose adjustments to CV medications. Nutritional interventions should be individualised according to nutritional status, frailty burden, comorbidities, body composition phenotype, and the presence of documented nutritional deficiencies rather than applied routinely to all older adults with CVD.

### 7.2. Micronutrient and Functional-Food Strategies

Nutrition should be embedded in comprehensive cardiac rehabilitation programmes. Diet-plus-exercise interventions yield greater improvements in VO_2_ peak and muscle strength than either modality alone. Geriatric rehabilitation teams can tailor meal timing around physical training to enhance protein utilisation (“anabolic window”). In older adults with HFpEF, combining a hypocaloric Mediterranean diet with aerobic exercise improved diastolic parameters and quality of life in the SECRET II trial [127].

Targeted micronutrient correction is critical. Thiamine repletion (100 mg/day) benefits patients on chronic diuretics; oral iron supplementation or intravenous ferric carboxymaltose may correct iron deficiency, enhance exercise tolerance, and maintain bone integrity and neuromuscular performance, thereby indirectly reducing falls [128,129]. Polyphenol-rich foods, berries, olive oil, and green tea exert antioxidant and anti-inflammatory effects relevant to vascular ageing. Emerging evidence supports the role of probiotics and prebiotics in restoring the gut microbiota and lowering circulating TMAO levels, potentially improving endothelial function [130,131]. When oral nutritional supplementation is considered, patient selection remains crucial. The available evidence suggests the greatest benefit in individuals with established malnutrition, nutritional risk, sarcopenia, frailty, or cardiac cachexia, particularly when supplementation is combined with physical rehabilitation or exercise-based programmes [132].

### 7.3. Addressing Polypharmacy and Socioeconomic Barriers

Polypharmacy frequently interferes with appetite or nutrient absorption, such as metformin with B12, proton-pump inhibitors with magnesium, and loop diuretics with thiamine [133]. Regular medication review is essential to minimise nutritional adverse effects. Social determinants such as poverty and isolation also impact food security; community-based services (home-delivered meals and food-voucher programmes) mitigate these barriers and should be integrated into discharge planning [134]. Socioeconomic and cultural factors may substantially influence nutritional status, dietary adherence, and access to CV care in older adults. Limited income, low educational level, social isolation, reduced mobility, and food insecurity may impair access to healthy dietary patterns, including Mediterranean-style nutrition, despite their potential CV benefit. Cultural dietary habits, health literacy, and caregiver support may further affect long-term adherence to nutritional recommendations [135]. These issues are particularly relevant in frail older adults with multimorbidity, cognitive impairment, or limited access to multidisciplinary care. Therefore, nutritional management in geriatric cardiology should incorporate not only biological and clinical variables but also broader social determinants of health and individualised barriers to care.

### 7.4. Digital and Tele-Nutrition Approaches

Digital and telehealth-based nutritional support strategies may improve adherence and long-term monitoring among selected older CV patients, although evidence remains limited [136,137,138]. Emerging digital health tools may further improve nutritional monitoring and functional assessment in older patients with CVD [137,139,140,141]. Smartphone applications designed for dietary tracking, hydration monitoring, medication adherence, and calorie or protein intake estimation may support self-management and reinforce long-term adherence to nutritional recommendations. In parallel, wearable technologies, including smartwatches and activity trackers, enable continuous monitoring of physical activity, walking distance, heart rate, sleep quality, and sedentary behaviour, parameters closely linked to frailty progression and CV prognosis in older adults [142,143].

**Figure 5:**
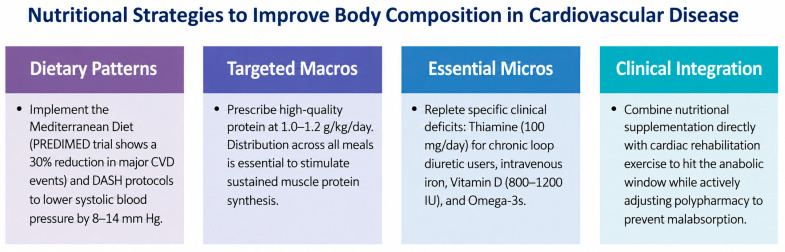
This figure outlines a multidimensional nutritional strategy aimed at improving body composition rather than focusing solely on weight reduction in patients with CVD. The framework integrates four complementary domains. First, evidence-based dietary patterns, such as the Mediterranean and DASH diets, support CV health and help control blood pressure. Second, targeted macronutrient intake, particularly adequate high-quality protein distributed across meals, helps preserve skeletal muscle mass and sustain muscle protein synthesis, which is particularly relevant in older or frail patients. Third, correction of key micronutrient deficiencies, including thiamine in patients receiving chronic loop diuretics, iron deficiency, vitamin D insufficiency, and inadequate omega-3 intake, represents an essential component of metabolic and CV optimisation. Finally, nutritional interventions should be integrated with structured cardiac rehabilitation and careful pharmacologic management in order to maximise anabolic recovery and reduce the risk of malabsorption or treatment-related nutritional deficits. This integrated approach may help interrupt the cycle linking malnutrition, sarcopenia, and adverse CV outcomes.

These technologies may facilitate earlier identification of functional decline, reduced mobility, or worsening nutritional status, particularly in patients with HF, frailty, or sarcopenia [143]. Integration of remote nutritional monitoring with telemedicine and cardiac rehabilitation programmes could also improve continuity of care after hospital discharge. However, digital interventions in older populations should account for cognitive status, digital literacy, visual impairment, caregiver support, and socioeconomic barriers, which may significantly influence adherence and feasibility [138,144]. A multidimensional approach integrating diet, micronutrient correction, rehabilitation, and pharmacologic optimisation is illustrated in Figure 5.

## 8. Future Directions

Research priorities include randomised trials specifically targeting frail elders that combine nutritional, physical, and pharmacologic interventions. Biomarkers such as prealbumin, phase angle, and inflammatory cytokines could help monitor response. Precision nutrition using metabolomic and microbiome profiling may identify individuals who derive maximal CV benefit from specific dietary patterns [145].

Public health initiatives should promote food environments that support healthy ageing, subsidising fruits, vegetables, and omega-3–rich products while reducing consumption of processed foods. Integrating nutrition education into medical curricula and cardiology training remains essential to overcome therapeutic nihilism toward dietary care in advanced age. Although a substantial body of literature supports an association between nutritional status and CV outcomes in older adults, much of the available evidence is derived from observational studies [146]. Therefore, caution is warranted when interpreting these findings as causal relationships. Future randomised controlled trials are needed to determine whether targeted nutritional interventions can directly improve CV outcomes, functional capacity, frailty trajectories, and quality of life in older patients with CVD [147].

## 9. Limitations

Despite the structured literature search and methodological approach adopted in this review, some limitations should be acknowledged. First, the available evidence derives from heterogeneous study designs, including observational studies, post hoc analyses, narrative reviews, and randomised clinical trials with variable representation of older and frail individuals. Second, substantial variability exists across studies in the definitions of malnutrition, sarcopenia, frailty, and obesity, as well as in the nutritional assessment tools and body composition techniques employed, limiting direct comparability of findings. Furthermore, many available studies were not specifically designed for older adults with CVD, and older adults with multimorbidity or severe frailty remain under-represented in clinical research. Although a structured search strategy and predefined eligibility criteria were applied, this review did not aim to conduct a quantitative meta-analysis; therefore, the synthesis of evidence retains some of the intrinsic limitations of a narrative review format, including potential selection and publication bias. These limitations highlight the need for larger prospective studies specifically focused on nutritional phenotyping and intervention strategies in geriatric CV populations.

## 10. Conclusions

Nutritional status is a modifiable determinant of CV health in the older adults. Both malnutrition and overnutrition accelerate vascular ageing through oxidative stress, inflammation, and metabolic dysregulation. Comprehensive assessment and early intervention can reverse this trajectory, improving function, quality of life, and survival. The synergy of balanced macronutrient intake, adequate protein, antioxidant micronutrients, and adherence to Mediterranean-type diets constitutes the foundation of “nutritional cardiology.” In the era of multimorbidity, integrating dietitians into HF and prevention clinics should be considered the standard of care for older adults. Nutritional phenotyping and multidimensional assessment should become integral components of precision CV care in older adults. Furthermore, dietary recommendations should be individualised according to frailty burden, cardiovascular phenotype, renal function, hydration status, and multimorbidity. In particular, sodium and fluid prescriptions should be tailored to the individual patient, avoiding overly restrictive approaches that may increase the risk of dehydration, orthostatic symptoms, sarcopenia, or functional decline in vulnerable older adults.

## Figures and Tables

**Table 1:** Clinical characteristics of major nutritional and functional phenotypes in older adults with CVD.

Phenotype	Clinical Description	Typical Findings	Common Assessment Approaches	Clinical Relevance
Malnutrition [21,22,23,24]	Condition resulting from inadequate intake, absorption, or utilisation of nutrients, leading to impaired body composition and functional status	Weight loss, reduced food intake, low albumin levels, and nutritional deficiencies	MNA, MNA-SF, GNRI, CONUT, PNI, and SGA	Associated with longer hospital stay, higher complication rates, and increased mortality
Cachexia [25,26]	Complex wasting syndrome occurring in the setting of chronic disease and characterised by ongoing loss of body weight and skeletal muscle mass	Involuntary weight loss, anorexia, systemic inflammation, and muscle depletion	Clinical evaluation, weight trajectory, inflammatory markers, and body composition assessment	Common in advanced HF and associated with poor prognosis
Sarcopenia [27,28]	Progressive decline in muscle mass accompanied by reduced muscle strength and physical performance	Reduced handgrip strength, slower gait speed, and impaired mobility	Handgrip strength, gait speed testing, BIA, DXA, and EWGSOP2 criteria	Increases the risk of falls, disability, hospitalisation, and mortality
Frailty [29,30]	State of increased vulnerability resulting from a decline in physiological reserve across multiple organ systems	Weakness, fatigue, reduced physical activity, and impaired resilience to stressors	Fried phenotype, Frailty Index, and Clinical Frailty Scale	Predicts adverse outcomes, loss of independence, and rehospitalisation
Sarcopenic obesity [31,32]	Presence of excess adiposity together with reduced muscle mass and/or muscle function	Visceral adiposity, reduced physical performance, and metabolic abnormalities	Body composition analysis combined with muscle function assessment	Associated with higher cardiometabolic risk and worse functional outcomes

Abbreviations: HF: Heart Failure; MNA: Mini Nutritional Assessment; MNA-SF: Mini Nutritional Assessment Short Form; GNRI: Geriatric Nutritional Risk Index; CONUT: Controlling Nutritional Status; PNI: Prognostic Nutritional Index; SGA: Subjective Global Assessment; BIA: Bioelectrical Impedance Analysis; DXA: Dual-Energy X-ray Absorptiometry; EWGSOP2: European Working Group on Sarcopenia in Older People 2.

**Table 2:** Main characteristics of the studies included in the qualitative synthesis.

Study	CV Setting	Nutritional Domain	Main Value
Knobloch 2025 [34]	Heart failure	Malnutrition	Prevalence/outcomes meta-analysis
Habaybeh 2021 [35]	Heart failure	Nutritional intervention	ONS/cachexia evidence
Belqaid 2024 [36]	Frail HF	Nutritional care	Frail older adults HF intervention review
González-Sosa 2024 [37]	Very old adults HF	Malnutrition/frailty	Prognosis in very old outpatients
Verdú-Rotellar 2024 [38]	Advanced HF	Malnutrition/QoL	QoL, function, and mortality
Abe 2024 [39]	Decompensated HF	Malnutrition + frailty	Combined phenotype predicts mortality
Calleja 2019 [40]	Atrial fibrillation	Sarcopenia/frailty	Sarcopenia predicts mortality
Cheng 2019 [41]	Very old adults AF	CONUT/PNI/GNRI	Nutritional indices predict events
Popiołek-Kalisz 2023 [42]	CAD	BMI/BIA/nutritional risk	Body composition and CAD severity
Vandewoude 2012 [43]	MSS	Sarcopenia–malnutrition	Methodological/background
Tonet 2020 [44]	ACS	MNA-SF	Mortality and physical performance
Tutumlu 2026 [45]	CAD older adults	MNA-SF/QoL	Malnutrition and quality of life
Lara-Breitinger 2021 [46]	Cardiac rehab	Nutritional care	Dietitian-led CR strategies
Kocanda 2023 [47]	Cardiac rehab	Nutrition intervention	Systematic review of CR interventions

**Table 3:** Age-related physiological and nutritional changes relevant to CV health.

System/Function	Age-Related Change	Nutritional Consequence	CV Impact
**Muscle**	↓ Mass and strength (sarcopenia)	Protein–energy deficit	Reduced cardiac output reserve
**Adipose tissue**	↑ Visceral fat and inflammatory cytokines	Insulin resistance	Atherosclerosis and HFpEF
**Endocrine**	↓ GH, IGF-1, and sex hormones	↓ Anabolism	Endothelial dysfunction
**Gastrointestinal**	↓ Acid, enzymes, and dysbiosis	Malabsorption	Micronutrient deficiency
**Kidney**	↓ GFR and concentrating ability	Dehydration and electrolyte loss	Volume instability and arrhythmia

Abbr. GH: Growth Hormone; IGF-1: Insulin-like Growth Factor 1; GFR: Glomerular Filtration Rate; HFpEF: Heart Failure with Preserved Ejection Fraction. The upward arrow (↑) indicates an increase, whereas the downward arrow (↓) indicates a decrease.

**Table 4:** Nutritional screening and assessment tools in the older adults.

Tool	Components	Cut-Off/Interpretation	Strengths/Limitations
**MNA**	Anthropometry, diet, and function	<17 = malnourished	Quick and validated for elders
**GNRI**	Albumin + weight ratio	<92 = high risk	Objective and uses labs
**PNI**	Albumin + lymphocytes	<45 = poor prognosis	Predicts surgical/CV outcomes
**SGA**	History + exam	Categories A–C	Clinician dependent
**BIA/DXA**	Body composition	Phase angle < 5° = cellular frailty	Quantitative but costlier

Abbr.: MNA: Mini Nutritional Assessment; GNRI: Geriatric Nutritional Risk Index; PNI: Prognostic Nutritional Index; SGA: Subjective Global Assessment; BIA: Bioelectrical Impedance Analysis; DXA: Dual-Energy X-ray Absorptiometry.

**Table 5:** Evidence-based dietary strategies for CV prevention in older adults.

Dietary Model	Key Features	Quantitative Benefit	Practical Notes
Mediterranean	High MUFA, fibre, and polyphenols	↓ CVD events ≈ 30% (PREDIMED)	Adapt portions to appetite
DASH	High K^+^, Ca^2+^, and Mg^2+^; low Na^+^	↓ SBP 8–14 mm Hg	Monitor renal function; sodium restriction should be individualised according to frailty status, HF phenotype, CKD, dehydration risk, orthostatic hypotension, and diuretic therapy
High-protein balanced	1.0–1.2 g/kg/day protein	↓ Frailty risk ≈ 25%	Combine with exercise
Omega-3 enriched	EPA + DHA 1–2 g/day	↓ TG 20–30%, anti-arrhythmic	Consume fish 2×/week
Plant forward	Legumes, soy, and whole grains	↓ LDL 10–15%	Supplement B12 if needed

Abbr.: MUFA: Monounsaturated Fatty Acids; K^+^: Potassium; Ca^2+^: Calcium; Mg^2+^: Magnesium; EPA: Eicosapentaenoic Acid; DHA: Docosahexaenoic Acid; TG: Triglycerides; LDL: Low-Density Lipoprotein; CVD: Cardiovascular Disease; SBP: Systolic Blood Pressure. ↓ means, reduction of.

## Data Availability

No new datasets were generated or analysed during the current study. All data discussed in this review were derived from previously published studies cited in the manuscript.
